# UN resolution on the elimination of harmful practices and the protection of human rights of mothers impacted by albinism

**DOI:** 10.7189/jogh.12.03029

**Published:** 2022-06-08

**Authors:** Sheryl Reimer-Kirkham, Ikponwosa Ero, Barbara Astle, Meghann Buyco, Emma Strobell

**Affiliations:** 1 Trinity Western University, Langley, British Columbia, Canada; 2 Under the Same Sun, Surrey, British Columbia, Canada

A recent United Nations (UN) resolution, The Elimination of Harmful Practices related to Accusations of Witchcraft and Ritual Attacks [1-3], was passed at the 47th session of the UN Human Rights Council (June 21 to July 13, 2021). The resolution states that “harmful practices related to witchcraft accusations and ritual attacks globally have resulted in various forms of violence, including killings, mutilation, burning, coercion in trafficking of persons, torture and other cruel, inhuman or degrading treatment and stigmatization” [1]. The resolution has far-reaching applications for those in vulnerable situations such as poverty and displacement, especially for women, children, older persons, persons with disabilities, and particularly persons with albinism.

By focusing on harmful practices, the resolution sidesteps, to some extent, the loaded construct of the term, witchcraft, and the associated challenges of academic and policy responses. Disciplinary scholarship (including anthropology, literature, legal studies, and health studies) diversely defines witchcraft, but there is a shared understanding that tends toward the belief in and use of supernatural or magical powers to achieve good or bad outcomes [4-7]. Mesaki, a Tanzanian anthropologist, points to the continuing salience of ideas about witchcraft in understanding contemporary African societies as “an idiom through which life is experienced and acted upon” [5], across urban/rural, elite/peasant, and rich/poor communities.

With its foregrounding of harmful practices, the resolution achieves a balance between the right to freedom of thought, conscience and religion on the one hand, and the limits to manifestations of these freedoms in order to protect the fundamental rights and freedoms of others on the other hand. The resolution also distinguishes traditional, complementary, and integrative medicine (as defined by the World Health Organization) from harmful practices related to accusations of witchcraft and ritual attacks that result in violations of human rights.

In relation to health, witchcraft and witchcraft accusations have been associated with conditions such as epilepsy, dementia, and psychosis, as exemplars of where cultural beliefs and practices carry profound impact. Persons with albinism, especially in some areas of Africa, have faced atrocious violations of their human rights, with abandonment, threats, mutilations and murder, purportedly for ritual practices and economic gain [6,8].

**Figure:**
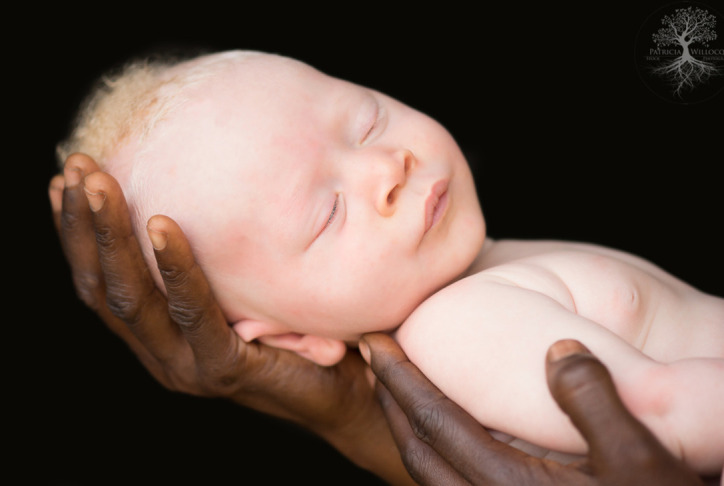
Photo: Persons with albinism, including their families, face stigma, discrimination and violence due to harmful practices related to witchcraft accusations in parts of Africa. Photo credit: Patricia Willocq, White Ebony Collection, with permission.

The relevance to mothers impacted by albinism – whether as women with albinism themselves, or as mothers of children with albinism – is that they carry a disproportionate burden on account of human rights violations. In our research with women in Tanzania and South Africa [9,10], accounts are widespread of dehumanizing discrimination, blame for the birth of a baby with albinism with subsequent abandonment by family and community, and their own constant surveillance to ensure the safety of their children with albinism. Although not the sole causative influence for human rights violations, harmful practices related to accusations of witchcraft and ritual attacks infuse the day-to-day realities of mothers impacted by albinism. Mothers reported being attacked or had witnessed both actual and/or threats of attacks on their children. For example, a mother described an attempted kidnapping of her 18-month-old child with albinism, plotted by the child’s own father and uncle [11]. Fortunately, a neighbour recognized the crying baby and drew attention to the man who had already cut off one of the child’s fingers. This left the mother fearing for the safety of her child, such that she was unable to go to work. She then made a difficult decision to send her child to a boarding school to ensure the child’s safety. Another mother who has albinism spoke about people’s responses of her delivering her child, reflecting how witchcraft accusations are associated with the birth of a baby with albinism: “Somebody put a witchcraft [curse] on me and that’s why my child is like this.” With such an attribution, she experienced name calling and discrimination from her community. The use of persons with albinism’s body parts for witchcraft practices, as well as the stigma and social exclusion suffered by mothers, contribute to harmful practices [12].This resolution, thus, carries immediate potential for the protection of their safety and security, by the line drawn between cultural practices (acceptable norms and practices), and those that are harmful and ought to be de-constructed as unacceptable due to elements that constitute harmful practices leading to human rights violations.

For the mothers in our study, the resolution provides alternative and empowering terminology for describing their experiences of albinism-related stigma. Given that the resolution is broadly affirming of cultural beliefs and practices, the mothers may, on this basis, describe their experiences in context of acceptable and non-acceptable cultural practices, and therefore become protagonists of favourable cultural dialogue and change. The mothers could use the resolution as a prevention measure or as an accountability measure. In the former instance, the resolution could serve as an authoritative instrument for the mothers to engage with their traditional and spiritual leaders – from local village chiefs to faith leaders – who tend to have significant influence in the community to identify normalized behaviours, that are in reality harmful practices, and how to counter them in their various constituencies. These normalized practices include name-calling and the propagation of misbeliefs about albinism including the belief that their body part can generate wealth when used in so-called witchcraft rituals. In the latter instance, the mothers could use the resolution as a basis to hold perpetrators accountable for those practices that are often not seen immediately as a crime with viable recourse. These harmful practices include abandonment of the child with albinism after birth and the forced cutting and sale of the hair of the child or woman with albinism by their parent or spouse. It is important that these cases be immediately brought to the attention of authorities – cultural or civic – as wrongdoing in the form of harmful practices, and as criminal offences where applicable. Undoubtedly, the above proposed use of the resolution will require targeted capacity development for the mothers including the vernacularization [13] of the text of the resolution, with translation into their local language as well as scenario training to illustrate and support its concrete application in their lives.

Future research should consider the incorporation of terminology in the resolution when gathering data on albinism particularly where negative myths and stigma on the condition are well documented. For legislators and policymakers, the resolution provides a crucial example of how to respond to violations related to witchcraft and ritual attacks without a consensus on the meaning of these terms.

People who, individually or with others, act to promote or protect human rights as activists and human rights defenders [14] would do well to raise awareness about this resolution and how it may facilitate cultural or normative change for the ultimate protection of people with albinism and their family members. Influential community leaders, such as faith leaders, should be educated about this resolution, and how their position can bring awareness to how harmful practices contravene the sacred teachings of their traditions. For example, our team recently facilitated a summit in Tanzania to bring together interreligious faith leaders who drafted a national statement on protecting the human rights and welfare of persons with albinism, with calls to action to government, faith leaders, community members, and persons with albinism [15]. Given the range in capacity and resource among human rights defenders, the better resourced defender-organizations should invest in extensive training workshops and capacity development at the grassroots level to engage relatively less-resourced community organizations such as the mothers’ support groups, on the content and significance of the resolution. In this training, emphasis should be placed on how to reframe and report normalized harm such as name-calling and blaming of spouses for having a child with albinism, using the nomenclature of harmful practices. This sensitization on the conceptual shift from normalized to harmful practice will, no doubt, take time. However, this shift will, in the end, contribute to deterrence and promote, in particular for mothers impacted by albinism, a deeper understanding of their human rights and their corresponding entitlements.
